# Supplementary material for: Mechanism of centromere recruitment of the CENP-A chaperone HJURP and its implications for centromere licensing
Source: Nat Commun. 2019 Sep 6;10:4046. doi: 10.1038/s41467-019-12019-6 (PMC6731319; doi:10.1038/s41467-019-12019-6)
Supplement: Supplementary file 1 — Supplementary Information [file 41467_2019_12019_MOESM1_ESM.pdf]

Supplementary information

Mechanism of centromere recruitment of the CENP-A chaperone HJURP and its implications for centromere licensing

Dongqing Pan, Kai Walstein, Annika Take, David Bier, Nadine Kaiser & Andrea Musacchio

Including Supplementary Figures 1 to 8.

Supplementary Data 1 (a resource table), Supplementary Data 2 (details of cross-links identified) and a Source Data file are available as separate Excel files.

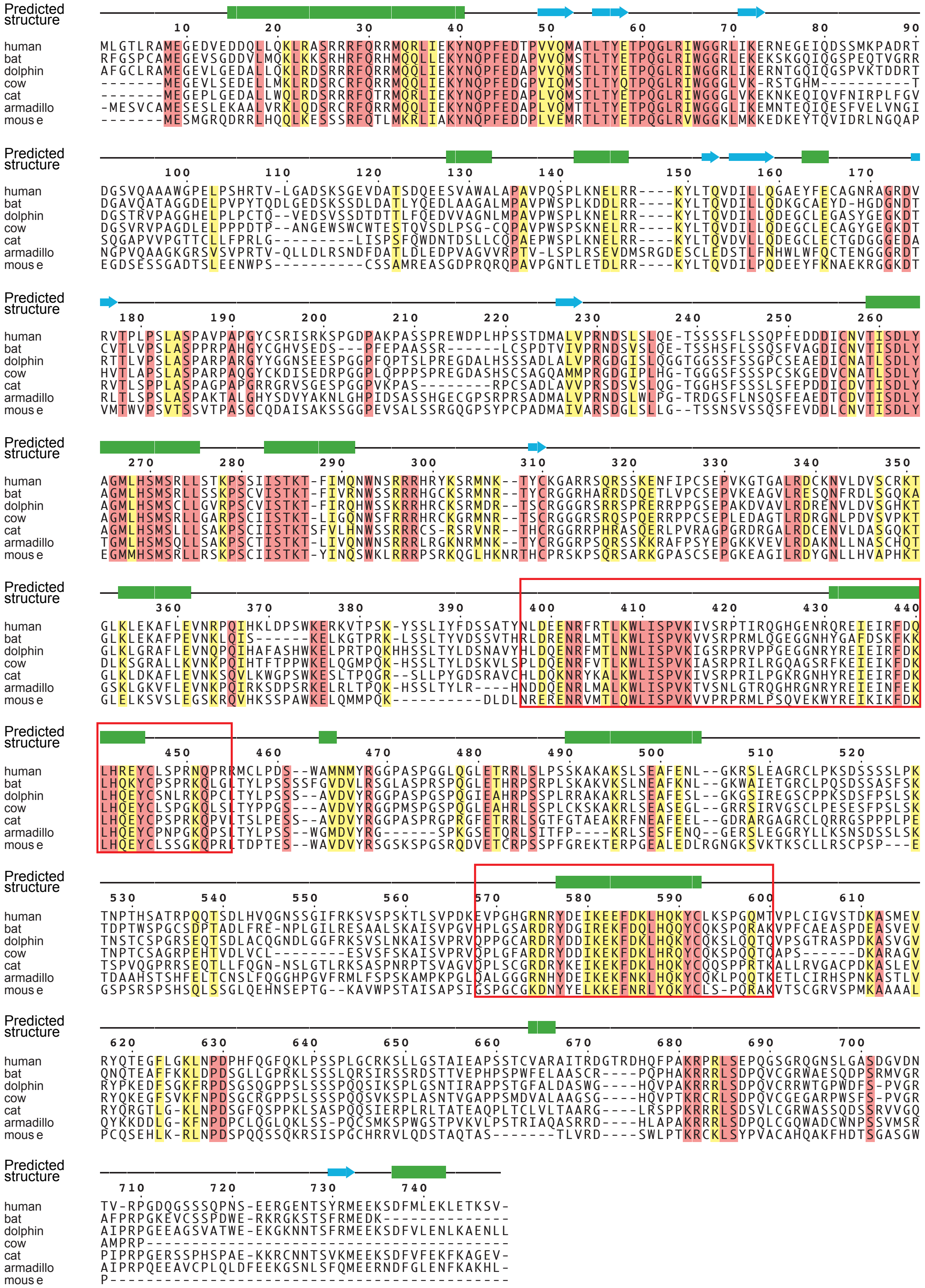

Supplementary Figure 1. Amino-acid sequence alignment of mammalian HJURP.
Multiple sequence alignment was generated using ClustalW (Larkin et al. 2007). Residues that are identical in all sequences are shaded red, and residues that only have conserved substitutions with similar properties are shaded yellow. Secondary structure prediction was performed using PSIPRED (Buchan et al., 2013). Green bars indicate the residues predicted to be in α-helical conformation. Blue arrows indicate the residues predicted to be β-strand. Red boxes indicate the parts of this alignment presented in Figure 1G. The amino-acid sequences were retrieved from UniProt or Ensembl databases with following identifiers: human, Q8NCD3 (UniProt); bat, G1PWU4 (UniProt); dolphin, ENSTTRP00000004146 (Ensembl); cow, E1BB33 (UniProt); cat, ENSFCAP00000051395 (Ensembl); armadillo, ENSDNOP00000013322 (Ensembl); mouse, Q6PG16 (UniProt).

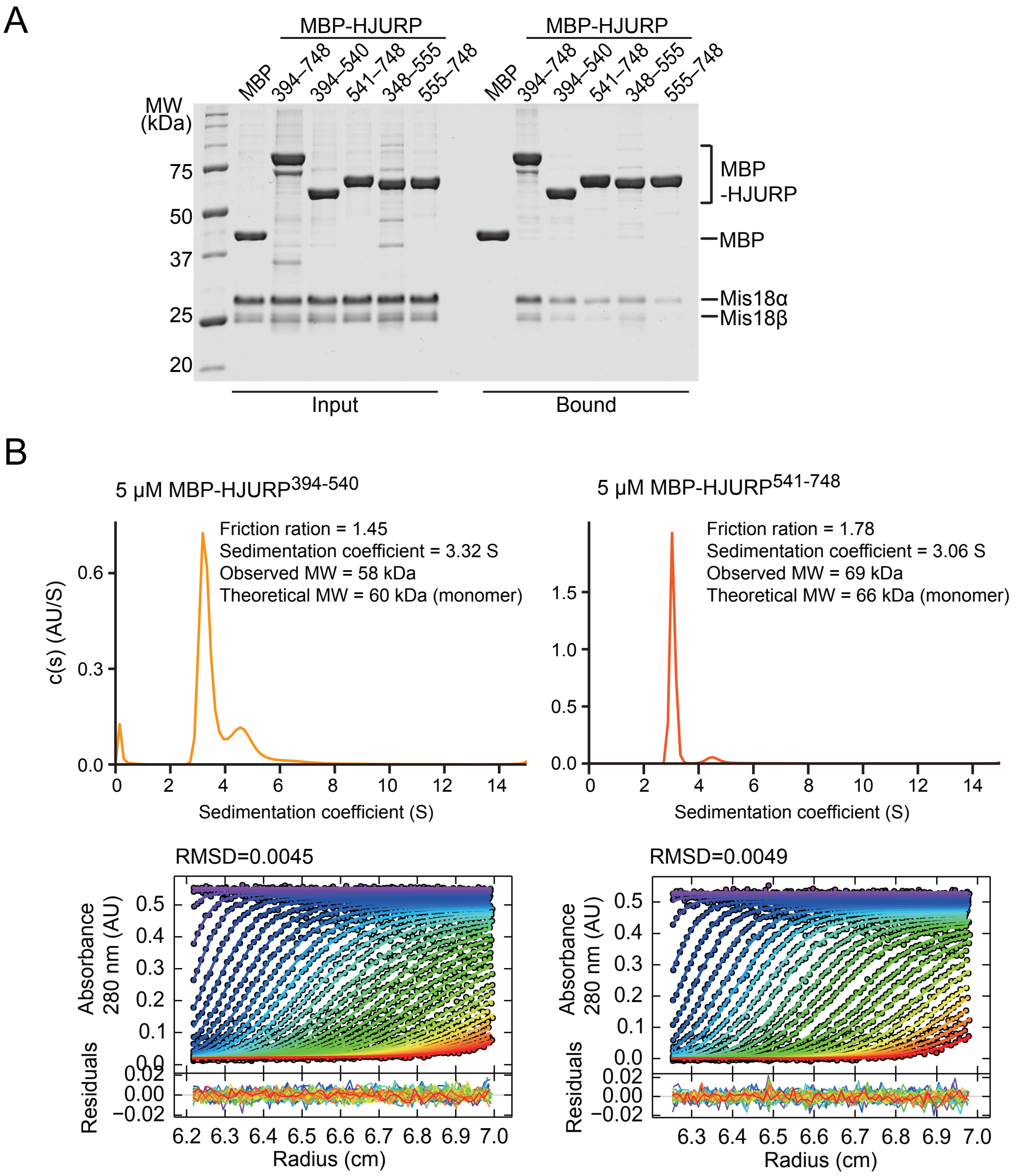

Supplementary Figure 2. Characterization of HJURP R1 and R2.
(A) Amylose-resin pull-down assays showing that HJURP$^{555–748}$ binds more weakly than HJURP$^{541-748}$ to Mis18^{core}. (B) Sedimentation velocity AUC results of MBP-HJURP$^{394–540}$ and MBP-HJURP$^{541-748}$.

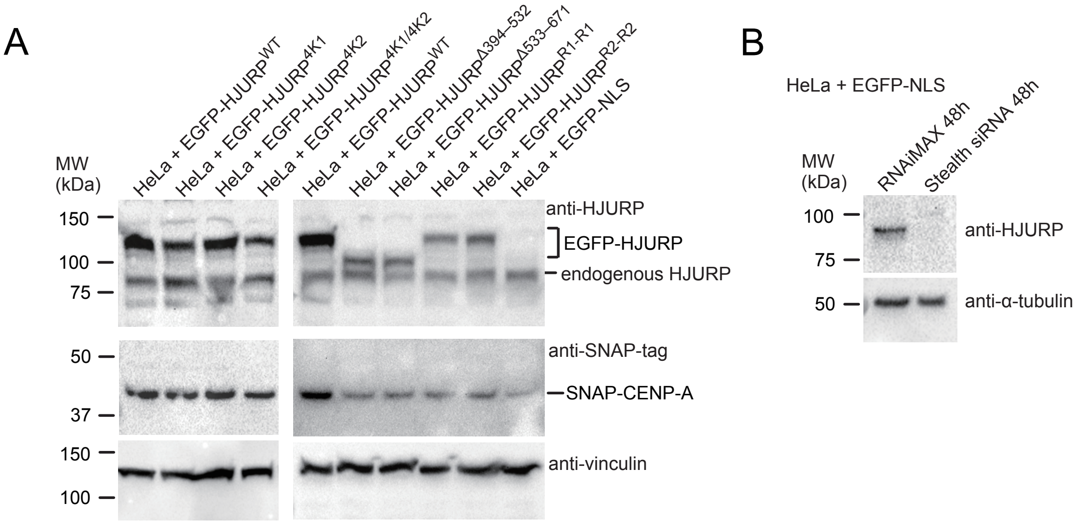

Supplementary Figure 3. Protein expression and HJURP depletion in HeLa cell lines.
(A) Western blots showing doxycycline-induced expression of EGFP-HJURP variants and SNAP-CENP-A in the stable HeLa cell lines used in this study. Cells were treated with 50 ng/mL doxycycline for 24 hours before harvesting. Clear lysate (containing 50 µg protein) obtained after cell disruption and centrifugation was applied on each lane of SDS-PAGE gels and blotted using the antibodies indicated in the figure. (B) Western blotting results showing the efficient depletion of the endogenous HJURP by the HJURP Stealth siRNA. Clear lysate containing 25 µg protein was analyzed for each condition. Source data are provided as a Source Data file.

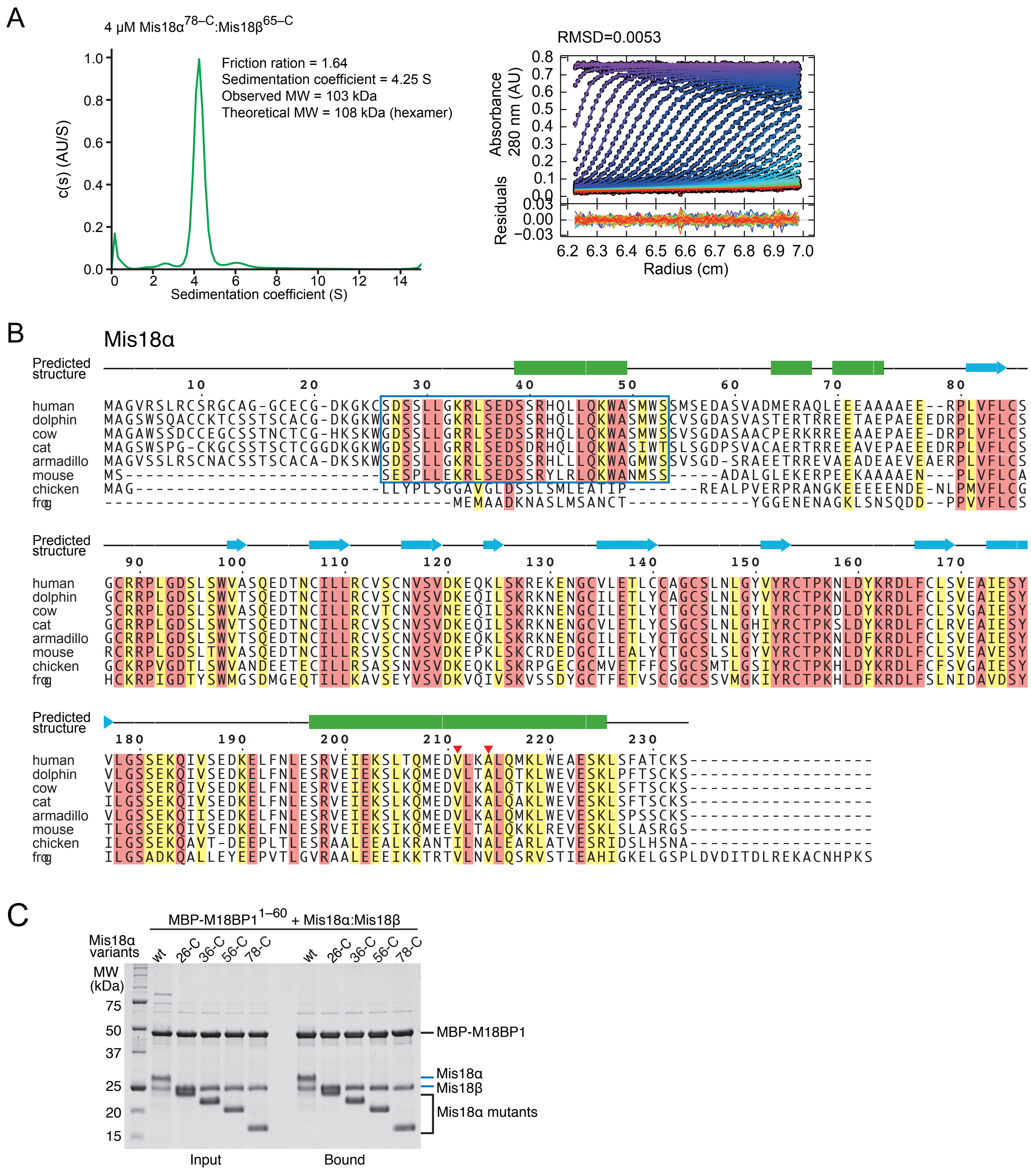

Supplementary Figure 4. Characterization of Mis18^{core} variants.

(A) Sedimentation velocity AUC results confirm the hexamer state of Mis18α^{78-C}:Mis18β^{65-C}. (B) Amino-acid sequence alignment of Mis18α. Residues that are identical in all sequences are shaded red, and residues that only have conserved substitutions with similar properties are shaded yellow. The residues in the boxed region (blue rectangle) are shaded in red and yellow according to their conservation among the mammalian species compared in this alignment. The amino-acid sequences were retrieved from UniProt, Ensembl or NCBI Protein databases with following identifiers: human, Q9NYP9 (UniProt); dolphin, A0A2U3V0K3 (UniProt); cow, A5D7N9 (UniProt); cat, M3WWJ6 (UniProt); armadillo, ENSDNOP00000004670 (Ensembl); mouse, Q9CZJ6 (UniProt); chicken, E1BQA3 (UniProt); frog, XP_002938488.2 (NCBI Protein). (C) Amylose-resin pull-down assays showing that deletion of the Mis18α N-terminal tail did not affect the interaction of Mis18^{core} with M18BP1$^{1-60}$.

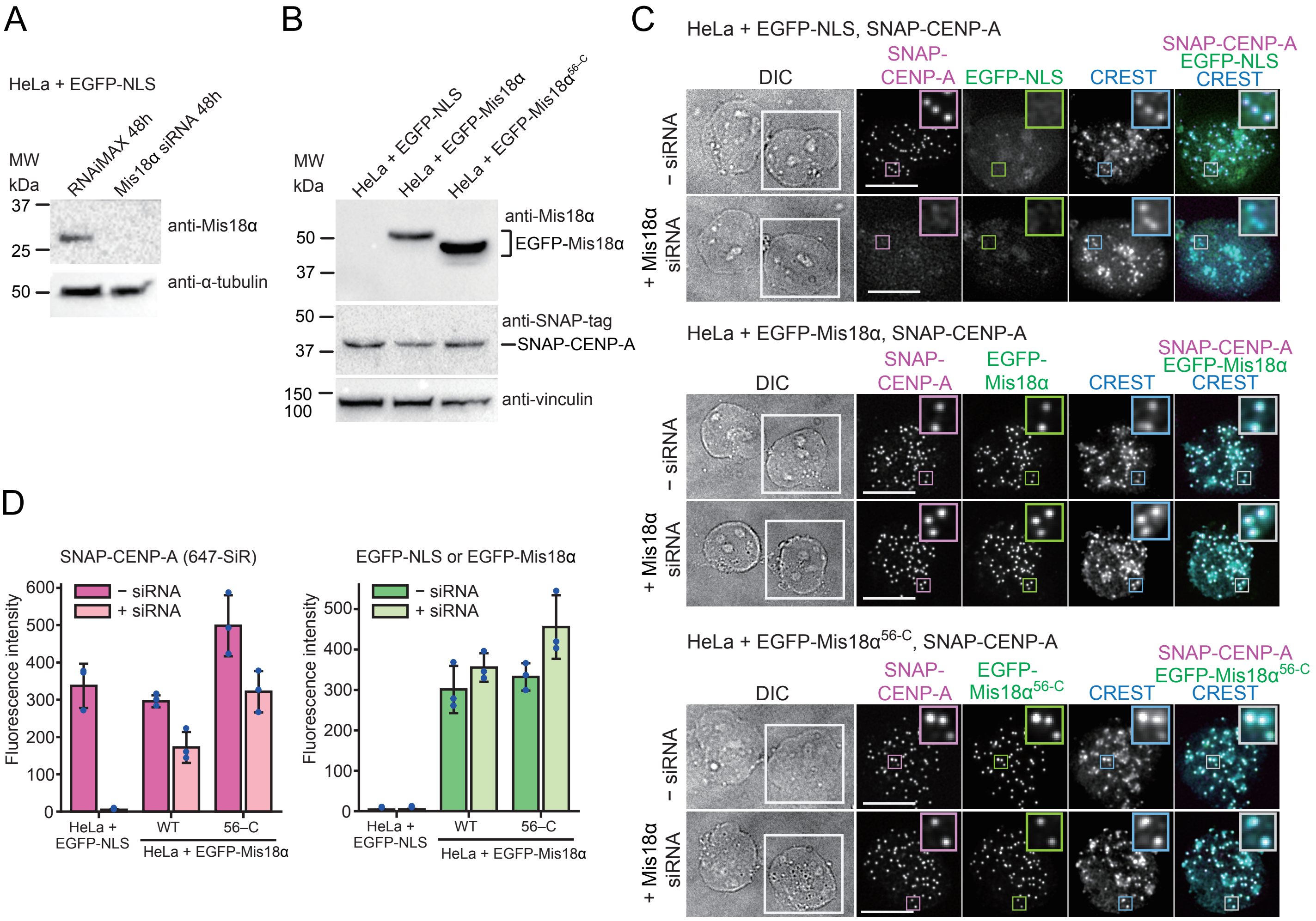

Supplementary Figure 5. Mis18α$^{56–C}$ mutant is functional for centromere localization and CENP-A deposition.
(A) Western blotting results showing the effective depletion of the endogenous Mis18α by Mis18α siRNA. Clear lysate containing 100 µg protein was analyzed for each condition. (B) Western blots showing doxycycline-induced expression of EGFP-Mis18α variants and SNAP-CENP-A in the stable HeLa cell lines used in this study. Clear lysate was prepared as described in the legend of Supplementary Fig. 3. Clear lysate containing 100 µg protein was analyzed by SDS-PAGE and western blotting using the antibodies indicated in the figure. (C) Representative images showing the fluorescence of SNAP-CENP-A and EGFP-NLS, EGFP-Mis18α or EGFP-Mis18α$^{56–C}$ in the fixed HeLa cells. CENP-A deposition experiments were performed as described in Figure 2A using Mis18α siRNA instead of HJURP siRNA. White scale bars indicate 10 µm. (D) Quantification of the centromere fluorescence intensity of SNAP-CENP-A and EGFP signals. The bar graphs represent mean values from three replicate experiments (blue dots indicate the mean values from each experiment). Error bars indicate standard deviations. Source data are provided as a Source Data file.

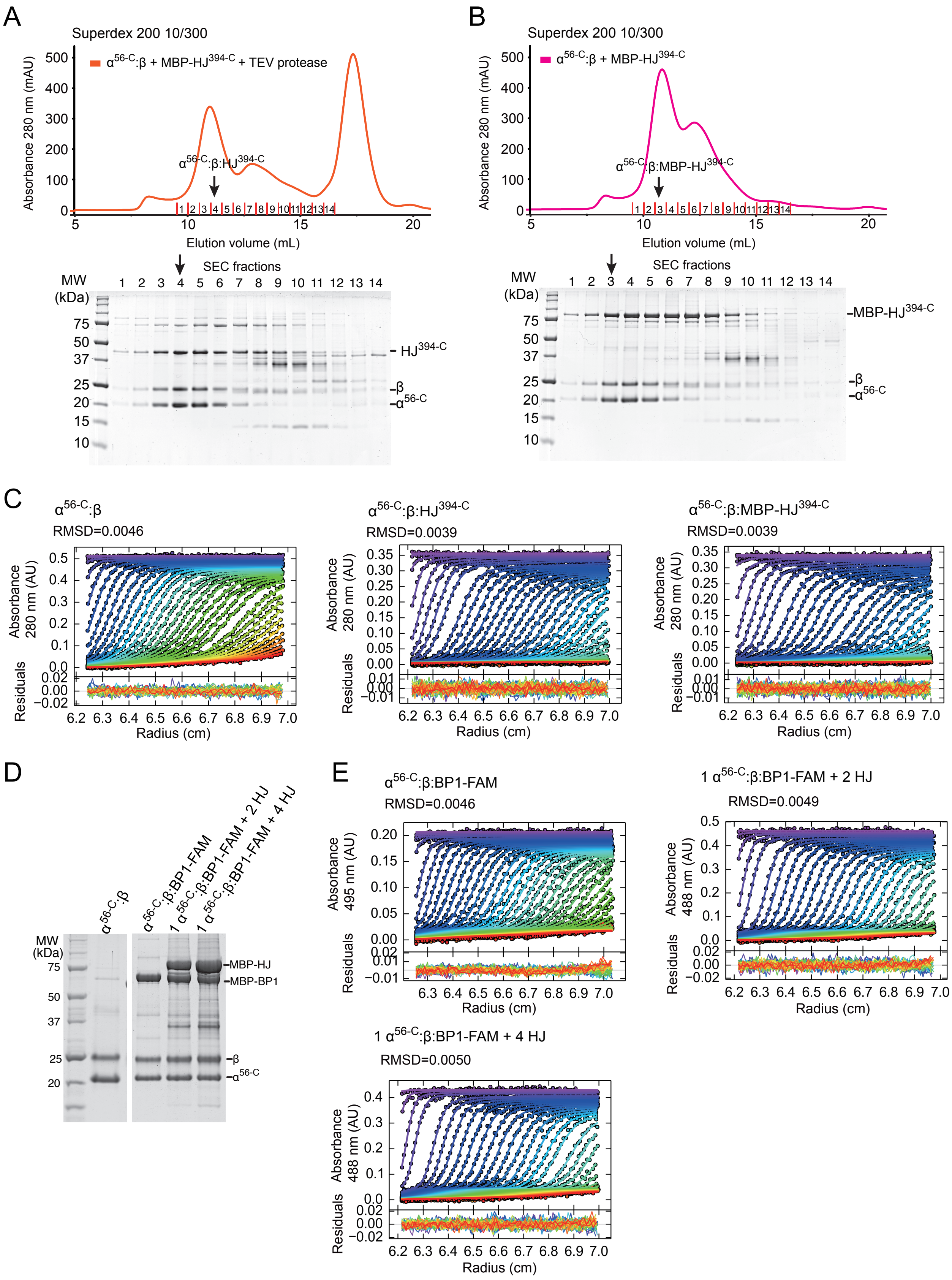

Supplementary Figure 6. HJURP forms a stable complex with Mis18^{core} and M18BP1

(A, B) SEC profiles and SDS-PAGE gels showing the results of sample preparation of Mis18α^{56-C}:Mis18β:HJURP^{394-C} complex or Mis18α^{56-C}:Mis18β:MBP-HJURP^{394-C} for AUC experiments. TEV protease was used to cleave off the MBP-tag from MBP-HJURP^{394-C} to obtain Mis18α^{56-C}:Mis18β:HJURP^{394-C} in (A). Black arrows indicate the SEC fractions from which samples were taken for AUC analyses presented in Figure 5G. (C) Best-fitting results of the sedimentation velocity AUC data of the AUC analyses presented in Figure 5G. (D) SDS-PAGE gels showing the samples used for the AUC analyses presented on Figure 6F. (E) Best-fitting results of the sedimentation velocity AUC data of the AUC analyses presented in Figure 6F. Source data are provided as a Source Data file.

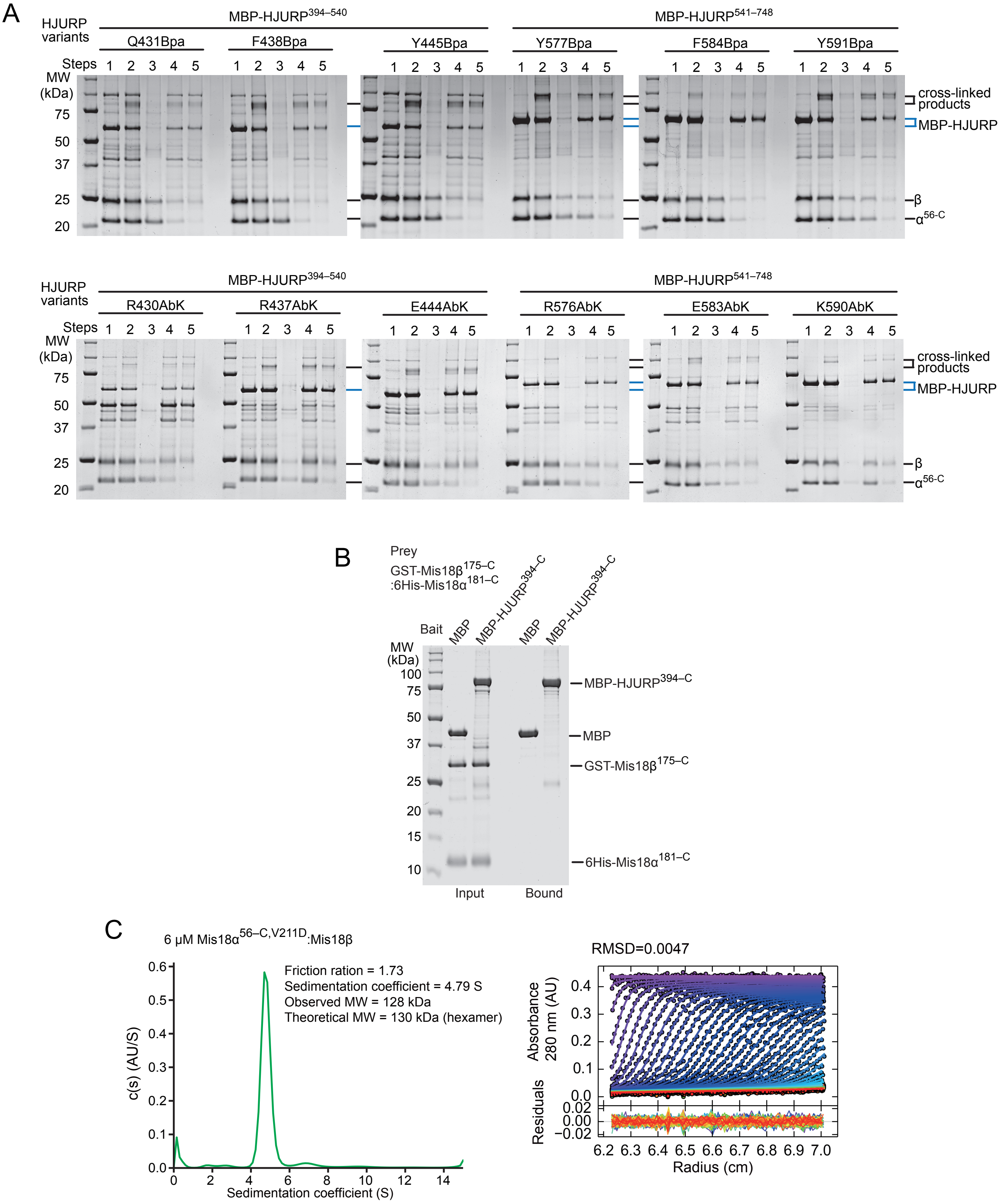

Supplementary Figure 7. UV-cross-linking experiments of HJURP incorporating Bpa or AbK and additional binding assays. (A) SDS-PAGE gel images showing the protein samples at each step of UV-cross-linking experiments. Step 1, Mis18α$^{56–C}$:Mis18β was incubated with MBP-HJURP fragments (with C-terminal 8His-tags) containing Bpa- or AbK-substitution at the indicated positions. Step 2, protein samples were exposed to UV light for 30 min. Step 3, unbound fraction of the Ni^{2+}-affinity purification was separated from the Ni^{2+}-resin. Step 4, Ni^{2+}-resin-bound fraction contained cross-linked products and free MBP-HJURP fragments and HJURP-bound Mis18α$^{56–C}$:Mis18β. Step 5, Ni^{2+}-resin-bound fraction after washing the beads with urea buffer contained less non-cross-linked Mis18α$^{56–C}$:Mis18β. (B) Amylose-resin pull-down assays showing that HJURP$^{394–C}$ does not pull-down the trimeric helix-bundle of Mis18α:Mis18β. Buffer B was used for the pull-down assays. (C) Sedimentation velocity AUC results of Mis18α$^{56–C,V211D}$:Mis18β.

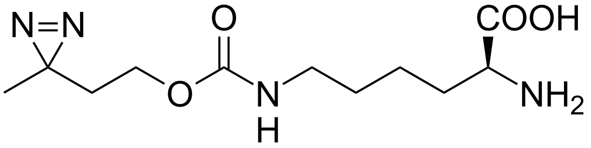

1

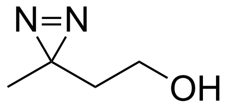

2

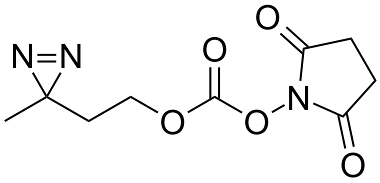

3

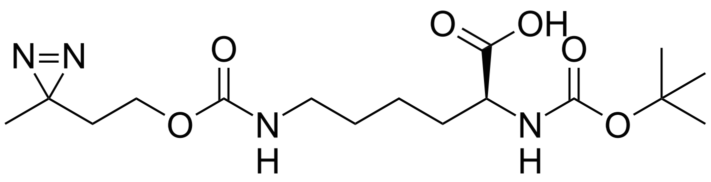

4

Supplementary Figure 8. Chemical structures related to the chemical synthesis of 3’-azibutyl-*N*-carbamoyl-lysine.
