# Supplementary material for: Mechanism of centromere recruitment of the CENP-A chaperone HJURP and its implications for centromere licensing
Source: Nat Commun. 2019 Sep 6;10:4046. doi: 10.1038/s41467-019-12019-6 (PMC6731319; doi:10.1038/s41467-019-12019-6)
Supplement: Supplementary file 3 — Description of Additional Supplementary Files [file 41467_2019_12019_MOESM3_ESM.pdf]

Description of Additional Supplementary Files

File Name: Supplementary Data 1
Description: **Resource Table.** The table reports type and source of plasmids, reagents (antibodies, chemicals, peptides, siRNA, primers), and cell lines (bacterial, mammalian) used in this study

File Name: Supplementary Data 2
Description: **Table of cross-links.** The table reports a list of inter-molecular crosslinks obtained by photo-activation of HJURP modified with Bpa at residues F438, Y445, Y557, F584, or Y591 or modified with AbK at residues E444, E583, or K590
