# Supplementary material for: Mechanism of centromere recruitment of the CENP-A chaperone HJURP and its implications for centromere licensing
Source: Nat Commun. 2019 Sep 6;10:4046. doi: 10.1038/s41467-019-12019-6 (PMC6731319; doi:10.1038/s41467-019-12019-6)
Supplement: Supplementary file 6 — Reporting Summary [file 41467_2019_12019_MOESM6_ESM.pdf]

nature research

Corresponding author(s): Andrea Musacchio, Dongqing Pan

Last updated by author(s): Aug 1, 2019

Reporting Summary

Nature Research wishes to improve the reproducibility of the work that we publish. This form provides structure for consistency and transparency in reporting. For further information on Nature Research policies, see Authors & Referees and the Editorial Policy Checklist.

Statistics

For all statistical analyses, confirm that the following items are present in the figure legend, table legend, main text, or Methods section.

n/a	Confirmed	
☐	☒	The exact sample size (*n*) for each experimental group/condition, given as a discrete number and unit of measurement
☐	☒	A statement on whether measurements were taken from distinct samples or whether the same sample was measured repeatedly
☒	☐	The statistical test(s) used AND whether they are one- or two-sided<br>*Only common tests should be described solely by name; describe more complex techniques in the Methods section.*
☒	☐	A description of all covariates tested
☒	☐	A description of any assumptions or corrections, such as tests of normality and adjustment for multiple comparisons
☐	☒	A full description of the statistical parameters including central tendency (e.g. means) or other basic estimates (e.g. regression coefficient) AND variation (e.g. standard deviation) or associated estimates of uncertainty (e.g. confidence intervals)
☒	☐	For null hypothesis testing, the test statistic (e.g. *F*, *t*, *r*) with confidence intervals, effect sizes, degrees of freedom and *P* value noted<br>*Give P values as exact values whenever suitable.*
☒	☐	For Bayesian analysis, information on the choice of priors and Markov chain Monte Carlo settings
☒	☐	For hierarchical and complex designs, identification of the appropriate level for tests and full reporting of outcomes
☒	☐	Estimates of effect sizes (e.g. Cohen's *d*, Pearson's *r*), indicating how they were calculated

Our web collection on statistics for biologists contains articles on many of the points above.

Software and code

Policy information about availability of computer code

Data collection	The program softWoRx (build 6.5.2) was used for the operation of a DeltaVision microscope and the data collection of cell images.
Data analysis	SEDFIT 14.4d was used for the analysis of AUC data. StavroX 3.6.6.6 was used for the analysis of MS data. Fiji (ImageJ version 2.0.0-rc-43/1.50e) was used for the analysis of cell images.

For manuscripts utilizing custom algorithms or software that are central to the research but not yet described in published literature, software must be made available to editors/reviewers. We strongly encourage code deposition in a community repository (e.g. GitHub). See the Nature Research guidelines for submitting code & software for further information.

Data

Policy information about availability of data

All manuscripts must include a data availability statement. This statement should provide the following information, where applicable:

- Accession codes, unique identifiers, or web links for publicly available datasets
- A list of figures that have associated raw data
- A description of any restrictions on data availability

The mass spectrometry data have been deposited to the ProteomeXchange Consortium via the PRIDE partner repository with the dataset identifier PXD013339.

Field-specific reporting

Please select the one below that is the best fit for your research. If you are not sure, read the appropriate sections before making your selection.

☒ Life sciences ☐ Behavioural & social sciences ☐ Ecological, evolutionary & environmental sciences

Life sciences study design

All studies must disclose on these points even when the disclosure is negative.

Sample size	Fluorescence intensity data were collected from more than 340 centromeres in each experiment, which is large enough for better estimation of the mean value.
Data exclusions	The highest 10% and the lowest 10% of fluorescence intensity data were excluded for the calculation of the mean values.
Replication	CENP-A loading experiments were performed at least three times. Pull-down assays were performed at least twice.
Randomization	Randomization was not applied to any experiments in this study.
Blinding	Blinding was not applied to any experiments in this study.

Reporting for specific materials, systems and methods

We require information from authors about some types of materials, experimental systems and methods used in many studies. Here, indicate whether each material, system or method listed is relevant to your study. If you are not sure if a list item applies to your research, read the appropriate section before selecting a response.

Materials & experimental systems

n/a	Involved in the study	
☐	☒	Antibodies
☐	☒	Eukaryotic cell lines
☒	☐	Palaeontology
☒	☐	Animals and other organisms
☒	☐	Human research participants
☒	☐	Clinical data

Methods

n/a	Involved in the study	
☒	☐	ChIP-seq
☒	☐	Flow cytometry
☒	☐	MRI-based neuroimaging

Antibodies

Antibodies used	Rabbit polyclonal anti-HJURP antibody (Abcam, ab100800), Rabbit polyclonal anti-Mis18α antibody (Thermo Fisher Scientific, PA5-53771), Rabbit polyclonal anti-SNAP-tag antibody (NEB, P9310), Mouse monoclonal anti-α-tubulin antibody (Sigma-Aldrich, T9026), Mouse monoclonal anti-vinculin antibody (Sigma-Aldrich, V9131), Sheep peroxidase-linked anti-mouse IgG (GE Healthcare, NXA931-1ML), Donkey peroxidase-linked anti-rabbit IgG (GE Healthcare, NA934-1ML), Human anti-centromere (CREST) (Antibodies Inc., 15-234-0001), DyLight 405-AffiniPure Donkey anti-Human IgG (Jackson ImmunoResearch, 709-475-149)
Validation	All primary antibodies used for western blotting detected proteins with expected sizes confirming the specific binding to the target proteins.

Eukaryotic cell lines

Policy information about cell lines

Cell line source(s)	All HeLa CENP-A-SNAP cell lines used in this study were generated from a Flp-In T-REx HeLa cell line generated by Stephen Taylor and colleagues, which we did not further authenticate.
Authentication	None of the cell lines used were authenticated.
Mycoplasma contamination	Cell lines were regularly tested for mycoplasma contamination.
Commonly misidentified lines (See ICLAC register)	We did not use any misidentified cell line.
